# Non-Coding RNA as Biomarkers and Their Role in the Pathogenesis of Gastric Cancer—A Narrative Review

**DOI:** 10.3390/ijms25105144

**Published:** 2024-05-09

**Authors:** Estera Bakinowska, Kajetan Kiełbowski, Patryk Skórka, Aleksandra Dach, Joanna Olejnik-Wojciechowska, Agata Szwedkowicz, Andrzej Pawlik

**Affiliations:** Department of Physiology, Pomeranian Medical University, 70-111 Szczecin, Poland; esterabakinowska@gmail.com (E.B.); kajetan.kielbowski@onet.pl (K.K.); p.skorka04@gmail.com (P.S.); dach.aleksandra@icloud.com (A.D.); olejnikjoanna25@gmail.com (J.O.-W.); szwedkowiczagata@gmail.com (A.S.)

**Keywords:** gastric cancer, non-coding RNA, microRNA, long non-coding RNA, circular RNA, biomarkers

## Abstract

Non-coding RNAs (ncRNAs) represent a broad family of molecules that regulate gene expression, including microRNAs, long non-coding RNAs and circular RNAs, amongst others. Dysregulated expression of ncRNAs alters gene expression, which is implicated in the pathogenesis of several malignancies and inflammatory diseases. Gastric cancer is the fifth most frequently diagnosed cancer and the fourth most common cause of cancer-related death. Studies have found that altered expression of ncRNAs may contribute to tumourigenesis through regulating proliferation, apoptosis, drug resistance and metastasis. This review describes the potential use of ncRNAs as diagnostic and prognostic biomarkers. Moreover, we discuss the involvement of ncRNAs in the pathogenesis of gastric cancer, including their interactions with the members of major signalling pathways.

## 1. Introduction

Gastric cancer (GC) is the fifth most frequently diagnosed cancer and the fourth most common cause of cancer-related death [1]. Based on the molecular profile, GC can be subdivided into gnomically stable, chromosomally unstable, microsatellite unstable and Epstein–Barr virus positive [2]. Examining the molecular profile of tumours introduced a significant breakthrough in the field of oncology, as it allows for the introduction of targeted therapies. For example, patients with GC overexpressing human epidermal growth factor receptor 2 (HER2) can be treated with a directed monoclonal antibody, trastuzumab [2,3]. Furthermore, multiple other targets are being examined as potential candidates for the treatment of GC [4]. Unfortunately, many patients with GC are diagnosed at advanced stages of the disease. Therefore, investigating strategies for early detection of GC could allow patients to be treated earlier. Moreover, the identification of biomarkers that could discriminate between treatment responders and non-responders, as well as factors associated with the prognosis, could improve long-term treatment outcomes. Currently, the commonly used and standardised tumour markers are tumour antigens and ectopic hormones [5]. In this review, we discuss the potential use of non-coding RNAs (ncRNAs) as diagnostic and prognostic biomarkers and their involvement in the pathogenesis of GC.

## 2. An Overview of Non-Coding RNA

The ncRNA family comprises a broad group of RNAs that participate in basic cellular functions, such as transfer RNA (tRNA) and ribosomal RNA (rRNA), as well as molecules that have received intense research attention in recent years. These ncRNAs are involved in a broad interaction network that regulates gene expression. ncRNAs are subdivided into small ncRNAs, including micro RNAs (miRNAs) and small interfering RNAs (siRNAs), as well as long non-coding RNAs (lncRNAs) [6]. The latter group includes circular RNAs (circRNAs), which are stable molecules with covalently closed loops [7]. The biogenesis of these molecules has been broadly discussed in several articles [8,9]. ncRNAs regulate gene expression in several ways. Classically, miRNAs bind to the 3′-untranslated region (UTR) of messenger RNA (mRNA) to suppress translation. On the other hand, lncRNAs and circRNAs can suppress miRNAs and act as sponges or competing endogenous RNAs (ceRNAs) to promote translation. The number of human lncRNAs is estimated to be over 100,000, and these molecules can be divided into three groups: transcribed from sequences complementary to protein-coding genes, known as natural antisense transcripts (NATS); transcribed from gene introns, known as intronic RNAs (incRNAs); and long intergenic non-coding RNAs (lincRNAs) that are located between genes [10]. Because ncRNAs impact gene expression, their dysregulation is involved in the pathogenesis of various inflammatory diseases and malignancies. In tumours, ncRNAs can silence tumour suppressors and promote the expression of oncogenes, ultimately leading to cancer progression. Several reviews have broadly discussed the roles of ncRNAs in the pathogenesis of cancer [11,12,13]. These RNAs can regulate the proliferation and apoptosis of cancer cells, as well as the metastatic potential or chemoresistance. Interestingly, ncRNAs can be used to detect or monitor disease progression, treatment or recurrence.

## 3. Non-Coding RNA in Gastric Cancer—Their Role as Biomarkers and Their Involvement in the Pathogenesis

### 3.1. MicroRNAs

#### 3.1.1. The Use of MicroRNAs as Diagnostic Biomarkers

miRNAs can be derived from various sources, including cancer tissue, blood and other biological fluids such as gastric digestive fluid. Molecules derived from GC tissue could help to establish a diagnosis or even suggest the specific cancer subtype. Importantly, large-scale databases such as The Cancer Genome Atlas (TCGA) have been created to improve our understanding of the genetic background of cancer formation. Regarding ncRNAs, multiple studies have analysed the TCGA database to study differentially expressed molecules that could be used as biomarkers or be implicated in the pathogenesis of GC. These analyses allow ncRNA expression in large cohorts to be studied. For example, using the TCGA collection, Pewarchuk et al. discovered 170 novel miRNAs in GC [14]. The detection of cancerous markers from patient blood is known as a liquid biopsy, which offers a non-invasive approach to search for molecules associated with cancer. Several studies have demonstrated that miRNAs can be identified in the blood, and their expression can be used in the diagnostic process. Some investigators have evaluated the diagnostic efficacy of RNAs located in exosomes, which are membrane-bound vesicles secreted by cells to communicate with each other by transferring proteins, lipids and/or nucleic acids. Tian et al. [15] found elevated expression of miR-181 and miR-652 in patients with GC compared with controls. The authors demonstrated that these molecules were upregulated in advanced GC compared with early cancer. The area under curve (AUC) of these molecules was 0.82 and 0.84, respectively [15]. The following miRNAs have also been suggested as potential biomarkers: miR-381; miR-107; miR-194; miR-210; miR-21; miR-196a-1; miR-146b; miR-17; miR-181a-1; miR-1-2; miR-139; miR-133b; miR-133a-2; miR-148a-3p; miR-21; miR-19a-3p; miR-483-5p; miR-191; miR-106a; miR-212; miR-143-3p; miR-515-3p; miR-940; miR-199a-3p; miR-383; miR-1236-3p; miR-551b-3p; and miR-551b-5p (Table 1) [16,17,18,19,20,21,22,23,24,25,26,27,28,29,30,31,32,33,34,35,36,37,38]. Interesting results can be obtained when large databases are integrated. For instance, higher expression of miR-17 in GC tissues was observed based on the TCGA database analysis. Using the Gene Expression Omnibus, elevated expression of miR-17 in GC tissues was found in five out of eight datasets. However, a reduced expression of the molecule was observed in three datasets when the sample type was blood [39]. Moreover, miRNAs encapsulated in exosomes can also be evaluated. Shi et al. [40] demonstrated that exosomal miR-1246 is upregulated in patients with GC and has great diagnostic potential. The abundance of these studies highlights the need to establish novel diagnostic markers for GC and the interest towards miRNAs in this matter.

RNAs could be used in the diagnosis of early disease [16], as well as specific subtypes of GC, such as the intestinal type [17]. Importantly, some researchers have found that miRNAs could have greater strength and diagnostic potential than classic GC markers, such as carcinoembryonic antigen (CEA) and carbohydrate antigen 19.9 (CA19-9) [26,38]. Furthermore, diagnostic tests could examine a panel of molecules to increase its efficacy. miRNAs could be combined with each other, different biochemical parameters as well as with other diagnostic methods, such as radiological imaging. For example, Huang et al. [41] combined six miRNAs that are differentially expressed in the serum of patients with GC and demonstrated that this combination enhanced the diagnostic potential compared with individual molecules. Another study showed that a panel composed of five miRNAs had higher diagnostic efficacy than CEA and CA19-9 [42]. In addition, a panel composed of miR-627, miR-629 and miR-652 showed very promising results in distinguishing patients with cancer from controls [43]. In an extensive analysis involving a cohort of 4566 patients, So et al. [44] demonstrated that an assay with 12 miRNAs effectively discriminated between patients with GC and controls.

The combination of miR-181 and miR-652 with the tumour marker CA72-4 demonstrated a higher diagnostic value (AUC of 0.917) compared with single molecules [15]. A combined evaluation of miR-6807-5p, miR-6856-5p and the *Helicobacter pylori* infection status also improved the AUC compared with the individual miRNAs [45]. Furthermore, a combination of miRNA and its target mRNA could increase diagnostic efficacy. Chen et al. [18] demonstrated the diagnostic potential of tissue-derived miR-139/FOS and miR-181a-1/KAT2B. However, in the validation cohort where patient plasma was examined, only miR-181a-1/KAT2B showed promising results. Han et al. [46] combined miR-135 and miR-20a with computed tomography (CT) and demonstrated that this test had higher accuracy, specificity and sensitivity compared with individual analyses of each of these methods. Similarly, a combination of miR-19a-3p and miR-483-5p with age also showed enhanced diagnostic potential [21]. As mentioned previously, miRNAs can be derived from sources other than tumour tissue and blood. For GC, several miRNAs in the gastric juice have been suggested as potential biomarkers [47,48,49].

**Table 1:** A summary of selected studies that have investigated the role of microRNAs (miRNAs) as diagnostic biomarkers of gastric cancer.

miRNA	Source	Expression/Concentration in Gastric Cancer	Diagnostic Potential (AUC)	Reference
miR-181 miR-652	Serum	Upregulated	miR-181: 0.82 miR-652: 0.84 Combined miR-181, miR-652, CA72-4: 0.917	[15]
miR-381	Serum	Downregulated	healthy vs. EGC: 0.922 EGC vs. AGC: 0.931	[16]
miR-107	Plasma	Downregulated	0.947	[17]
miR-194		Downregulated	0.862	
miR-210		Downregulated	0.82	
miR-1246	Serum	Upregulated	0.95	[40]
miR-21	Tissue	Upregulated	0.993	[18]
miR-196a-1		Upregulated	0.948	
miR-146b		Upregulated	0.935	
miR-17		Upregulated	0.909	
miR-181a-1		Upregulated	0.931	
miR-1-2		Downregulated	0.903	
miR-139		Downregulated	0.930	
miR-133b		Downregulated	0.909	
miR-133a-2		Downregulated	0.905	
miR-181a-a/KAT2B	Tissue and Plasma	-	Tissue: 0.96 Plasma: >0.95	
miR-148a-3p	Plasma	Downregulated	0.83	[19]
miR-21	Blood	Upregulated	0.8	[20]
miR-135	Serum	Upregulated	0.873	[46]
miR-20a	Serum	Upregulated	0.793	
miR-19a-3p	Serum	Upregulated	0.77	[21]
miR-483-5p	Serum	Upregulated	0.758	
miR-191	Serum	Upregulated	0.85	[22]
miR-106a	Plasma	Upregulated	0.895	[23]
miR-212	Serum	Downregulated	0.96	[24]
miR-10b-5p, miR-132-3p, miR-185-5p, miR-195-5p, miR-20a-3p, miR-296-5p	Serum	Upregulated	0.703	[41]
miR-143-3p	Plasma	Downregulated	0.9156	[25]
miR-21, miR-31, miR-92a, miR-181b, miR-203	Serum	Different expression depending on the molecule	0.9	[42]
miR-515-3p	Serum	Upregulated	0.8555	[26]
miR-940	Plasma	Downregulated	0.9657	[27]
miR-199a-3p	Plasma	Upregulated	0.837	[28]
miR-627, miR-629, miR-652	Plasma	Upregulated	0.969	[43]
miR-383	Tissue	Downregulated	0.8	[29]
miR-1236-3p	Tissue	Downregulated	0.7016	[30]
miR-551b-3p	Serum	Downregulated	0.86	[31]
miR-551b-5p	Serum	Downregulated	0.84	[32]
miR-26a	Plasma	Downregulated	0.882	[33]
miR-223	Serum	Upregulated	0.85	[34]
miR-16	Serum	Upregulated	0.9	
miR-100	Serum	Upregulated	0.71	
miR-200c	Blood	Upregulated	0.715	[35]
miR-206	Serum	Downregulated	0.89	[36]
miR-222	Plasma	Upregulated	0.85	[37]
miR-421	Serum and PBMC	Upregulated	Serum: 0.779 PBMC: 0.821	[38]

AGC—advanced gastric cancer; EGC—early gastric cancer; PBMC—peripheral blood mononuclear cells.

Downregulated miRNA in serum may reflect the expression pattern in cancerous tissue and suggest a positive role of such molecules in tumourigenesis. miR-107 was downregulated in GC tissue compared with paracancerous tissue. Amongst potential targets, Yan et al. [50] identified that miR-107 inhibits TP53-regulated apoptosis inhibitor 1 (TRIAP1), which can enhance tumour progression. Therefore, suppressed miR-107 does not inhibit the pro-cancerous TRIAP1 in GC. However, for a particular molecule to become a diagnostic marker, its concentrations should change similarly in the general population. Furthermore, its role in the progression of GC should be investigated thoroughly. Unfortunately, in many cases, conflicting results regarding the role of miRNAs in the pathogenesis of different or even the same cancers have been published. In the case of miR-107, other studies have found that it is upregulated in patients with cancer, and its inhibition could be associated with beneficial outcomes [51,52,53].

Overall, the abovementioned studies have demonstrated an important role of miRNAs as potential diagnostic biomarkers of GC. However, these molecules can also point at populations with increased susceptibility to GC. Specifically, harbouring certain miRNA gene polymorphisms may predispose individuals to the development of cancer. Polymorphisms can change the binding affinity, expression or molecular structure of miRNAs, changes that can alter their biogenesis and/or ability to modulate gene expression. Jin and Yu [54] suggested that carriers of the G allele of the single nucleotide polymorphism (SNP) located at the +60 locus of miR-421 have an increased risk of developing GC. Furthermore, the C allele of the rs2620381 miR-627 SNP was significantly associated with GC [55]. Importantly, abundant studies have been published regarding the role of certain SNPs, and meta-analyses have been performed to increase the strength of the findings. Xie et al. [56] demonstrated that carriers of the GC genotype of the miR-146a rs2910164 SNP (vs. the GC genotype and vs. the GC + CC genotype) were significantly associated with the disease. Moreover, Rong et al. [57] performed a meta-analysis and included a total of 9745 controls and 3954 patients with GC. The authors demonstrated that the C allele of the mRNA-499 rs3746444 T>C SNP increased susceptibility to GC. Several other SNPs—miR-196a2 rs11614913, miR-27a rs895819, miR-499 rs3746444, miR-27a rs895819, miR-4427 rs701213, miR-548j rs4822739, miR-938 rs12416605, miR-4274 rs1553867776, miR-3175 rs1439619, miR-6891 rs6149511, miR-8084 rs404337, miR-4719 rs7500280 and miR-34b/c rs4938723—have been associated with an increased or decreased susceptibility to GC [58,59,60,61,62]. Interestingly, SNPs have been combined with other parameters to evaluate the correlation with gastric malignancy. Interestingly, the combination of the miR-196a2 rs11614913 polymorphism and *H. pylori* seems to be associated with gastric precancerous lesions [58].

As mentioned above, miRNA SNPs could alter the RNA molecular structure. Landeros et al. [61] showed that several polymorphisms alter miRNA structure. For example, the miR-6891 rs6149511:T>TGAAGGGCTCCA SNP significantly changes the loop terminal region. Such modification could impair the generation of mature forms of miRNAs. Moreover, examining several SNPs could also provide beneficial information. Specifically, Pan et al. [62] showed that a combination of certain genotypes of miR-34b/c rs4938723 and TP53 Arg72Pro SNPs could identify patients with reduced susceptibility to GC. Taken together, the abovementioned studies have shown that miRNAs can be used to identify populations with increased susceptibility to GC. Additionally, these molecules can be used as diagnostic biomarkers to detect early stages of the disease or to support the currently used diagnostic protocols.

#### 3.1.2. The Role of MicroRNAs in the Pathogenesis of Gastric Cancer and as Prognostic Biomarkers

As discussed in the previous section, numerous miRNAs are differentially expressed in patients with GC. Apart from diagnostic purposes, studies have examined their prognostic potential. Their effect on survival in patients with cancer depends on their interactions with signalling pathways. Tumourigenesis is a complex process that involves numerous signalling pathways. Their hyperactivation contributes to abnormal proliferation and suppression of apoptosis of malignant cells. miRNAs have been implicated in a broad interaction network and regulate the expression of numerous genes and pathways. Altered expression of miRNAs may stimulate oncogenes or silence tumour suppressors. These pathways involve phosphoinositide 3-kinase/Akt/mammalian target of rapamycin (PI3K/AKT/mTOR), mitogen-activated protein kinases (MAPKs) and Wnt/β-catenin, amongst others.

**Figure 1:**
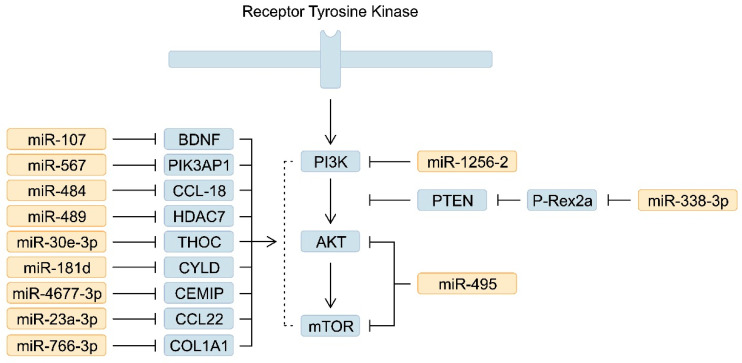
A simplified representation of the phosphoinositide 3-kinase/Akt/mammalian target of rapamycin (PI3K/Akt/mTOR) signalling pathway and the involvement of downregulated microRNAs (miRNAs) in its regulation.

The activity of PI3K/AKT/mTOR signalling is associated with GC tumourigenesis, as the expression of molecules that enhance this pathway is associated with poorer survival [63,64]. Furthermore, targeting the PI3K/AKT/mTOR signalling pathway has been suggested as a promising strategy for treating GC [65]. This pathway is regulated by numerous miRNAs, and their altered expression contributes to the activation or suppression of signalling. Consequently, modification of miRNA expression could represent another potential treatment strategy in GC. miR-567 is downregulated in GC tissues. It targets and suppresses the expression of PIK3AP1, a molecule that enhances phosphorylation of PI3K, AKT and c-Myc, a downstream element of the pathway [66]. Interestingly, miR-567 is also involved in mediating the properties of cancer stem cells (CSCs), an important population of tumour cells that drive resistance to chemotherapy and radiotherapy [67]. Similarly, downregulation of miR-107 is also associated with enhanced activity of the PI3K/Akt pathway. Mechanistically, miR-107 targets the brain-derived neurotrophic factor (BDNF) [68]. In addition, the downregulation of miR-4677-3p [69], miR-23a-3p [70], miR-495 [71], miR-181d [72], miR-30e-3p [73], miR-489 [74], miR-484 [75] and miR-125b-2 [76], miR-338-3p [77] and miR-766-3p [78], amongst others, enhances PI3K/Akt signalling (Figure 1). Importantly, these molecules can serve as prognostic factors, as they have been associated with overall survival (OS) [70,72,73,74], disease-free survival (DFS) [71] and progression-free survival (PFS) [74]. Furthermore, altered expression of RNAs may correlate with treatment response or be associated with a particular treatment agent. For example, reduced expression of miR-34a has been associated with cisplatin resistance [79]. miR-495 directly targets and inhibits the expression of Akt and mTOR. Therefore, its reduced expression might indicate a potential application as a PI3K inhibitor [71]. In another study, miR-107 was upregulated in GC tissues, and contrary to the previous findings, the molecule seemed to stimulate the PI3K/Akt pathway—the use of an miR-107 inhibitor suppressed tumour growth in an in vivo experiment [51]. Thus, depending on cellular context or other mechanisms, miRNAs may act as drivers of tumourigenesis, also known as onco-miR or tumour suppressors.

Numerous upregulated miRNAs also take part in promoting the activity of the PI3K/AKT/mTOR signalling pathway. miR-21 is one of the most extensively studied miRNAs. It is involved in the pathogenesis of several cancers [80,81,82]. In GC, miR-21 is upregulated. Furthermore, its enhanced expression significantly promotes the proliferation of malignant cells. Mechanistically, the molecule targets and inhibits phosphatase and tensin homolog (PTEN), a major tumour suppressor that inhibits PI3K/Akt signalling [83]. mir-21 is a downstream element of the signal transducer and activator of the transcription 3 (STAT3) pathway, also implicated in the pathogenesis of GC [84,85]. Unsurprisingly, miR-21 expression has been shown to correlate with clinical parameters such as DFS, OS, lymph node metastasis and tumour differentiation [86,87]. In addition, miR-21 is involved in mechanisms that confer treatment resistance. Specifically, there was higher expression of miR-21-5p in GC cells resistant to doxorubicin, and its silencing was associated with restoration of sensitivity [88]. There were similar results regarding the resistance of GC cells to cisplatin [89]. Interestingly, miR-21 was also shown to participate in the development of resistance to trastuzumab, the previously mentioned monoclonal antibody that targets HER2 [90]. miR-21 also has immunomodulatory features. As demonstrated by Zheng et al. [91], it is regulated by programmed cell death protein 1 (PD-1), and it regulates the balance between Th17 and regulatory T cells (Tregs). Therefore, studies have investigated whether miR-21 could be a target for GC treatment. Importantly, the use of an miR-21 inhibitor and short hairpin RNA (shRNA) was associated with decreased invasiveness of GC cells [92,93]. Moreover, the combination of anti-miR-21 with trastuzumab and 5-fluorouracil demonstrated important anticancer properties [94].

**Figure 2:**
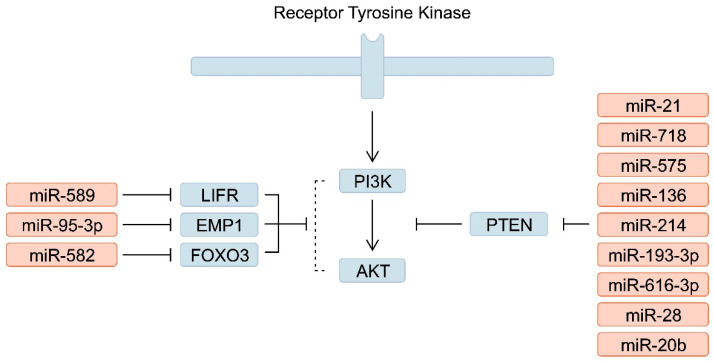
A simplified representation of the phosphoinositide 3-kinase (PI3K)/Akt signalling pathway and the involvement of upregulated microRNAs (miRNAs) in its regulation.

Other miRNAs upregulated in patients with GC that target PTEN include miR-718 [95], miR-575 [96], miR-136 [97], miR-214 [98], miR-193-3p [99], miR-616-3p [100], miR-28 [101] and miR-20b [102], amongst others. In addition, GC cells secrete miRNAs encapsulated in exosomes. Du et al. [103] demonstrated that GC cells could enhance angiogenesis by secreting extracellular vesicle-containing miR-23a that targets PTEN in endothelial cells. Importantly, miRNAs can regulate the expression of PTEN indirectly by binding to PTEN regulators. The expression of miR-32-5p has been elevated in GC tissues. The molecule targets and inhibits Krüppel-like factor 2 (KLF2), a transcription factor known to enhance PTEN expression; thus, miR-32-5p indirectly stimulates the PI3K/Akt pathway [104]. miR-589 [105], miR-95-3p [106] and miR-582 [107], amongst others, are upregulated in GC and activate the PI3K/Akt pathway by binding to other elements (Figure 2). These molecules have also been studied as potential prognostic factors associated with OS [95,96,100,106]. In addition, researchers have found associations with other clinical parameters. Specifically, miR-582 expression was significantly upregulated in patients with GC and lymph node, liver and lung metastases [107]. Moreover, due to their involvement in the signalling pathway, miRNAs have been associated with treatment resistance. For example, miR-95-3p has been suggested to drive cisplatin resistance in GC: this miRNA was upregulated in cisplatin-resistant cancer tissue [106].

MAPK pathways, including extracellular signal-regulated kinase (ERK)-MAPK, p38-MAPK and c-Jun N-terminal kinase (JNK)-MAPK, are also regulated by ncRNAs, and this activity could play a role in the pathogenesis of GC [108]. miR-95 is upregulated in GC and is associated with elevated expression of phosphorylated ERK and JNK [109]. Similarly, miR-574-5p could promote phosphorylation of ERK1/2 after transfection in GC cells. The molecule enhanced the expression of vascular endothelial growth factor A (VEGFA), which naturally enhanced angiogenesis [110]. Other miRNAs with dysregulated expression in GC cells or tissues and associated with the regulation of MAPK signalling include miR-181a-5p [111], miR-124-3p [112], miR-135b [113], miR-206 [114], miR-128 [115], miR-1271 [116], miR-141 [117], miR-204-3p [118] and miR-633 [119]. Apart from investigating the expression profile of miRNAs in GC, the precise mechanisms that underlie these alterations remain unknown. Nevertheless, epigenetic regulation may be associated with these observations. Specifically, gene methylation could dysregulate the expression of RNAs. Lim et al. [116] found 122 differentially methylated miRNAs in GC, including hypermethylation of miR-1271, a molecule downregulated in GC. Importantly, the authors also found that miR-1271 targeted and inhibited the expression of MEK1, a member of the MAPK cascade. Consequently, changing the methylation profile might reverse dysregulated expression of miRNAs and thus suppress tumour formation.

Similarly to the PI3K/Akt pathway, regulation of MAPK signalling also modulates drug resistance. For example, elevated miR-135b expression was associated with enhanced MAPK signalling. By contrast, reducing the expression of this miRNA increased the sensitivity of GC cells to cisplatin [113]. Introducing an miR-195 mimic, a molecule that inhibits the expression of MAPK3, was associated with enhanced apoptosis of cisplatin-resistant GC cells [114]. Importantly, the regulation of MAPK signalling is not only associated with the proliferation or migration of GC; it is also involved in immune escape. Malignant cells express checkpoint inhibitors, such as programmed death-ligand 1 (PD-L1), that suppress the activity of T cells. The expression of miR-675-3p in GC tissues was upregulated. This molecule targets CXXC finger protein 4 (CXXC4), a negative regulator of MAPK signalling. Consequently, the expression of PD-L1 was enhanced, and miR-675-3p stimulated the immune escape of GC through MAPK signalling [120]

Interestingly, certain molecules regulate both the PI3K/Akt and MAPK signalling cascades. For example, sprouty2 (Spry2) is a negative regulator of the abovementioned pathways, and its expression in GC tissues is reduced. miR-592 targets and inhibits Spry2, thus activating both signalling pathways. Importantly, higher expression of this miRNA was associated with lymph node metastasis, histological type and tumour size [121]. miR-338 also regulates the activity of both cascades. Peng et al. [122] demonstrated that its expression is reduced in GC tissues and cell lines. Through targeting neuropilin 1, overexpression of miR-338 could reduce phosphorylation of Akt, p38 and ERK1/2. Furthermore, as mentioned previously, GC tumours may overexpress HER2 and specific inhibitors of this receptor are used in clinical practice. Overexpression of HER2 also causes dysregulation of the MAPK and PI3K/Akt signalling cascades. Therefore, miRNAs targeting HER2 or its regulators might demonstrate beneficial effects in the treatment of HER2-dependent GC. Tokumaru et al. [123] investigated the use of synthetic miR-143 in GC cells. The authors found that the molecule targeted DEAD/H-box RNA helicase 6 (DDX6), a positive regulator of HER2. Consequently, synthetic miR-146 reduced the expression of HER2. An in vivo experiment demonstrated that an intravenous administration of miR-146 significantly suppressed tumour growth in mice.

Malignant cells can secrete ncRNA-containing exosomes that change the behaviour of cells in tumour microenvironment. Macrophages are one of several cellular subtypes present in the tumour microenvironment. These cells exhibit different phenotypes, each associated with specific features. The pro-inflammatory M1 and anti-inflammatory M2 macrophages are the two most commonly studied phenotypes. M2 macrophages create an environment that suppresses cytotoxic reactions in the tumour. Intriguingly, Qiu et al. [124] found that GC cells secrete exosomes containing miR-519a-3p, a molecule that enhances MAPK signalling in macrophages, driving their polarisation towards the M2 phenotype. miRNAs that regulate the activity of the MAPK signalling cascade can also be associated with prognosis. For example, elements/regulators of MAPK signalling are targeted by miR-142-5p and miR-375. Zhang et al. [125] demonstrated that combining these two molecules may help in evaluating the risk of recurrence in patients with GC who undergo surgery. Furthermore, the expression of these molecules, such as miR-141 and miR-633, has also been associated with lymph node metastasis [117,119].

The Wnt/β-catenin signalling pathway is also regulated by ncRNAs. Briefly, by binding to the protein complex composed of LRP5/6 and frizzled (Fz), Wnt ligands prevent ubiquitination of β-catenin, which allows the molecule to enter the nucleus and regulate gene expression [126]. Studies have demonstrated that aberrant activation of the Wnt/β-catenin cascade is associated with GC progression [127,128,129]. By modulating the activity of the pathway, miRNAs affect the progression of GC. For example, one study showed that miR-455-3p is downregulated in GC tissue. The miRNA inactivated the Wnt/β-catenin cascade, and miR-455-3p mimics enhanced apoptosis of GC cells and inhibited epithelial-to-mesenchymal transition [130]. There have been similar findings regarding miR-219-5p. Mechanistically, the molecule suppressed the expression of liver receptor homolog-1 (LRH-1), which regulates Wnt signalling activity [131]. miR-140-5p targets and inhibits the expression of Wnt1 and β-catenin, thus suppressing the invasiveness of GC cells. Similar to other molecules, miR-140-5p expression has been correlated with clinical parameters. Specifically, lower expression was associated with poorer OS and DFS, together with a more advanced TNM stage and lymph node metastasis [132]. miR-520f-3p is another downregulated miRNA that regulates Wnt signalling; it has been correlated with OS and DFS in patients with GC [133]. By contrast, several other miRNAs that regulate the Wnt pathway are upregulated in GC cells or tissues, including miR-324-3p [134], miR-194 [135], miR-192, miR-215 [136], miR-501-5p [137] and miR-483-5p [138], amongst others. Interestingly, the PI3K/Akt pathway is also associated with Wnt/β-catenin signalling. miR-188-5p upregulation enhanced the Wnt signalling cascade by targeting PTEN. Importantly, elevated expression of this miRNA was also correlated with lymph node metastasis and OS [139]. Table 2 summarises miRNAs associated with selected clinical parameters in GC.

GC tumourigenesis is a multifactorial and complex process. Microbial infections can contribute to tumour formation. *Helicobacter pylori* infection is the most widely studied pathogen that contributes to the formation of GC. Intriguingly, studies have found that pathogens also induce alterations in the ncRNA profile that may drive the development of malignancies. Specifically, approximately 40 dysregulated miRNAs were observed in stomach cells due to *H. pylori*, resulting in hundreds of differently expressed mRNAs [140]. Stimulation of GC cells with *H. pylori* significantly upregulated miR-183 expression, which was associated with reduced expression of FOXO1 [141]. The expression of miR-204 is also modified by *H. pylori*. Mechanistically, the molecule suppressed the activity of the nuclear factor kappa-light-chain-enhancer of activated B cells (NF-κB) pathway, a major inflammatory component. The pathogen could downregulate the expression of miR-204, thus enhancing the NF-κB signalling pathway [142]. Taken together, numerous miRNAs have been implicated in the pathogenesis of GC. These molecules regulate the activity of major signalling pathways that modulate the proliferation and apoptosis of cancer cells. Interestingly, agents that suppress GC tumour growth also affect the expression of miRNAs. For example, Wang et al. [143] demonstrated that salidroside, a metabolite of *Rhodiola*, arrested the cell cycle and enhanced apoptosis of GC cells. Furthermore, the authors found that the treatment stimulated the expression of the tumour suppressor miR-1343-3p.

### 3.2. Long Non-Coding RNAs—Involvement in the Pathogenesis of Gastric Cancer and Their Role as Biomarker

lncRNAs are composed of more than 200 nucleotides and are involved in several mechanisms regulating gene expression. In this article, we will mostly discuss lncRNAs that act as miRNA sponges. However, these molecules can also mediate transcription by regulating chromatin accessibility. For instance, lncRNAs act as scaffolds that can regulate histone modifications and gene expression. In addition, they interact with proteins to regulate splicing and signalling pathways [144,145].

Multiple studies have evaluated whether they can be used to detect GC. Elevated expression of the prostate cancer gene expression marker 1 (PCGEM1) in GC cells and tissue has been observed. Furthermore, higher plasma levels of this molecule were detected in patients with GC. The AUC of 0.750 indicated promising diagnostic efficacy. Importantly, it showed greater strength than other commonly used GC markers (CEA, CA12-5, CA72-4, AFP and CA19-9), but the combination of PCGEM1 with these markedly increased the efficacy (AUC = 0.815) [146].

Similarly, the diagnostic and prognostic potential of H19 [147] and highly upregulated in liver cancer (HULC) [148] have been evaluated. There were higher concentrations of these molecules in the patient’s serum. Moreover, their levels showed a significant correlation with GC. Zhou et al. [147] indicated a positive correlation between H19 and the GC stage. They used received operating characteristic (ROC) curve analysis to assess the discriminatory ability between early-stage cancer and controls. The AUC, sensitivity and specificity were 0.877, 0.855 and 0.801, respectively. Jin et al. [148] reported an AUC of 0.888 for HULC.

Another promising diagnostic biomarker is the Hox transcript antisense intergenic RNA (HOTAIR). HOX factors are crucial regulators of transcription, as they have the property of modifying the landscape of chromatin accessibility [149]. An increasing number of investigators have shown that HOTAIR overexpression may have a significant impact on GC tumour formation and progression. In their meta-analysis, Yang et al. [150] showed that HOTAIR may be associated with a worse prognosis for patients with GC and oesophageal cancer. Furthermore, the above study indicated that the elevated expression of this biomolecule was associated with shorter OS (hazard ratio [HR] 1.56, 95% confidence interval [CI] 1.38–1.75, *p* < 0.001). HOTAIR interacts with other molecules, such as miR-34a, which consequently activates the PI3K/AKT signalling pathway. In addition, it is associated with the proto-oncogenic MYC family, oestrogen response elements (ERE) and NF-κB, amongst others [151,152]. Measuring the HOTAIR level in plasma has been suggested as a promising method to diagnose GC. Specifically, Elsayed et al. [153] found that HOTAIR plasma levels provided a high sensitivity and specificity of 88% and 84%, respectively. Furthermore, increased expression was associated with higher grades and the presence of metastasis. In addition, its expression correlated positively with CEA levels (r = 0.426, *p* = 0.002), and the use of combined markers increased the AUC (0.954).

lnc-G protein subunit alpha Q-6:1 (lnc-GNAQ-6:1) and proprotein convertase subtilisin/kexin type 2-2:1 (PCSK2-2:1) have also been suggested to serve as diagnostic biomarkers. Patients with GC had significantly lower lnc-GNAQ-6:1 serum levels, and the sensitivity and specificity were 83.7% and 55.6%, respectively. In addition, the combination of CEA, CA19-9 or CA72-4 with lnc-GNAQ-6:1 had an AUC of 0.735, 0.757 and 0.776, respectively [154]. PCSK2-2:1 has also been found in serum exosomes and its concentrations were decreased in patients with GC (AUC = 0.896) [155]. In addition, PCKS2-2:1 expression correlated positively with tumour diameter (*p* = 0.0441), tumour stage (*p* = 0.0061) and the degree of venous invasion (*p* = 0.0367). Compared with the CEA, CA19-9 and CA724, PCKS2-2:1 demonstrated significantly greater diagnostic efficacy, with an AUC of 0.498, 0.541, 0.570 and 0.896, respectively.

In another study, the authors reported increased serum levels of zinc finger NFX1-type containing 1 antisense RNA 1 (ZNFX1-AS1) and HULC in patients with GC [156]. ROC analysis indicated an AUC of 0.850 for ZNFX-AS1 and 0.650 for HULC. In addition, these lncRNAs had a high diagnostic value compared with traditional serum biomarkers (CEA, CA19-9, CY211 and NSE). CEA had the highest AUC (0.620). In addition, ZNFX1-AS1 plasma levels correlated positively with lymphatic invasion; therefore, monitoring ZNFX1-AS1 levels might be useful in evaluating the prognosis after surgical treatment.

Dong et al. [157] examined a panel of three serum lncRNAs, including CUDR, long stress-induced non-coding transcript 5 (LSINCT-5) and phosphatase and tensin homolog pseudogene 1 (PTENP1). It showed promising diagnostic efficacy with an AUC of 0.829. By contrast, the AUC for CEA and CA19-9 was 0.529 and 0.591, respectively. Importantly, the AUC increased for the detection of patients with stage 1 cancer, suggesting a promising role of this panel in the detection of early-stage cancer.

Another interesting marker is the long non-coding actin filament-associated protein 1-antitense RNA 1 (AFAP-AS1). There was an increased expression of the lncRNA in tissues from patients with GC (AUC = 0.893) [158]. 

F-Box protein, helicase, 18-antisense RNA (FBXO18-AS) promotes transforming growth factor beta1 (TGF-β1) expression by activating TGF-β1/small mother against decapentaplegic (Smad) signalling. There was an increased expression of this biomarker in GC cells. Moreover, patients with high FBXO18-AS expression had a shorter OS compared with patients with low expression. Additionally, the AUC of 0.822 confirmed the diagnostic potential [159].

Moazzen et al. [160] showed that the BCL2 ovarian killer gene (BOK-AS1), family with sequence similarity 215 member A (FAM215A) and FEZF1 antisense RNA (FEZF1-AS1) are upregulated in GC tissue. The AUC was 0.737 for BOK-AS1, 0.716 for FAM215A and 0.712 for FEZF1-AS1 was 0.712.

An in vitro and in silico study indicated a high diagnostic value of the lncRNA small nucleolar RNA host gene15 (SNHG15). The AUC, specificity and sensitivity of SNHG15 were 0.744, 63.5% and 79.7%, respectively [161]. The above study, as well as the previous report by Chen et al. [162], clearly indicate that increased expression of these biomarkers may be useful in GC.

Li et al. [163] performed bioinformatics analysis to identify diagnostic and prognostic lncRNA and mRNA biomarkers for stomach adenocarcinoma. The authors identified 814 mRNAs and 106 lncRNAs from the TCGA dataset. They selected three diagnostically optimal lncRNAs, namely RP11-598F7.5, LINC01235 and FOXD2-AS1. ROC curve analysis showed that the AUC was 0.965 for FOXD2-AS1, 0.916 for LINC01235 and 0.936 for RP11-598F7.5. In addition, high LINC01235 expression correlated positively with shorter survival of patients with gastric adenocarcinoma (*p* = 0.012). The three biomarkers showed 122 lncRNA–mRNA co-expression pairs, indicating the diagnostic potential of combining lncRNAs with their target mRNAs. Table 3 summarises the diagnostic potential of selected lncRNAs.

Interestingly, the diagnostic potential of lncRNAs may be combined with magnetic resonance imaging (MRI). As reported by Chen et al. [164], MRI-based radiomics is a valuable tool for assessing the clinical prognosis and diagnosis of advanced GC, as indicated by the AUC for 3-year (0.690) and 5-year (0.687) OS. Perhaps the combination of this method with previously described markers could improve the detection of GC. The radiogenomics-based study by Gao et al. [165] suggested that CT could be employed in patients with GC by using appropriate gene models to detect direct blood vessel invasion. The combination of lncRNA and CT could be used to assess prognosis.

**Figure 3:**
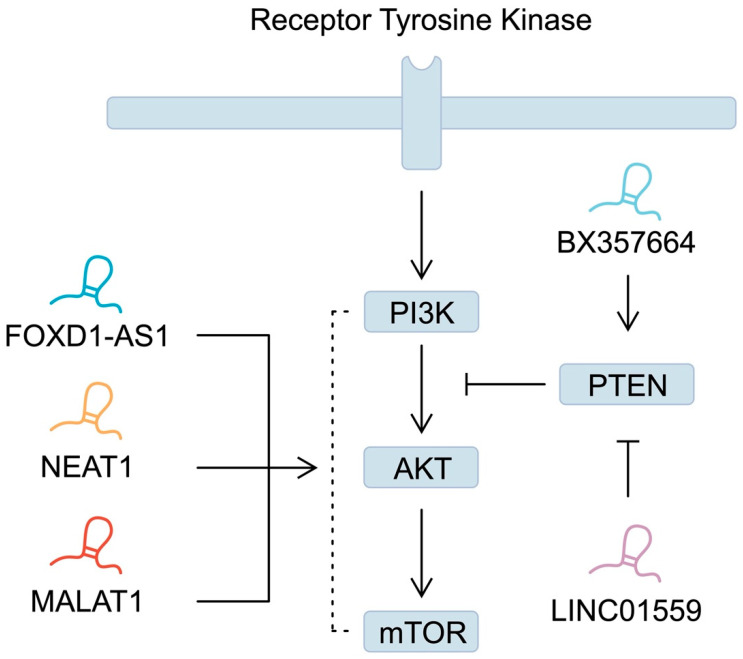
A simplified representation of the phosphoinositide 3-kinase/Akt/mammalian target of the rapamycin (PI3K/Akt/mTOR) signalling pathway and the involvement of long non-coding RNA (lncRNA).

Similarly to miRNAs, dysregulated expression of lncRNAs disrupts signalling pathways. As reported by Wu et al. [166], forkhead box D1 antisense RNA 1 (FOXD1-AS1) promotes GC progression and chemoresistance by activating the PI3K/AKT/mTOR pathway. Mechanistically, it sponges miR-466, which leads to the upregulation of PIK3CA. Similarly, nuclear-enriched abundant transcript 1 (NEAT1) interacts with miR-1294 to stimulate the expression of AKT1 [167]. In addition, lncRNAs mediate the expression of PTEN, the previously mentioned negative regulator of the PI3K/AKT pathway. For instance, BX357664 is downregulated in GC tissues. Its overexpression was associated with suppressed GC tumorigenesis and elevated expression of PTEN. The observed effect on the signalling pathway was exerted by sponging miR-183-3p [168]. By contrast, lncRNAs can also suppress the activity of PTEN. Wang and colleagues demonstrated that LINC01559 is downregulated in GC tissues based on the TCGA and the Gene Expression Profiling Interactive Analysis databases. As previously mentioned, lncRNAs are involved in other mechanisms apart from sponging miRNAs. In this study, the authors demonstrated that LINC01559 could recruit the enhancer of zeste 2 polycomb repressive complex 2 subunit (EZH2), an enzyme mediating the epigenetic process of methylation. In this case, recruited EZH2 could methylate the PTEN promoter to suppress its expression [169]. In addition, the PI3K/AKT pathway is regulated by more widely studied lncRNAs, such as metastasis-associated lung adenocarcinoma transcript 1 (MALAT1). Its expression in GC tissues is upregulated, which was demonstrated based on the TCGA database analysis, as well as verifications performed on smaller samples. High expression of this lncRNA is correlated with poorer survival. Mechanistically, studies have demonstrated that MALAT1 can activate PI3K/AKT signalling, which has been associated with cisplatin resistance [170,171] (Figure 3). YiRen and collaborators suggested that resistance to treatment could be the consequence of the impact of MALAT1 on autophagy and interaction with miR-23b-3p [172].

**Figure 4:**
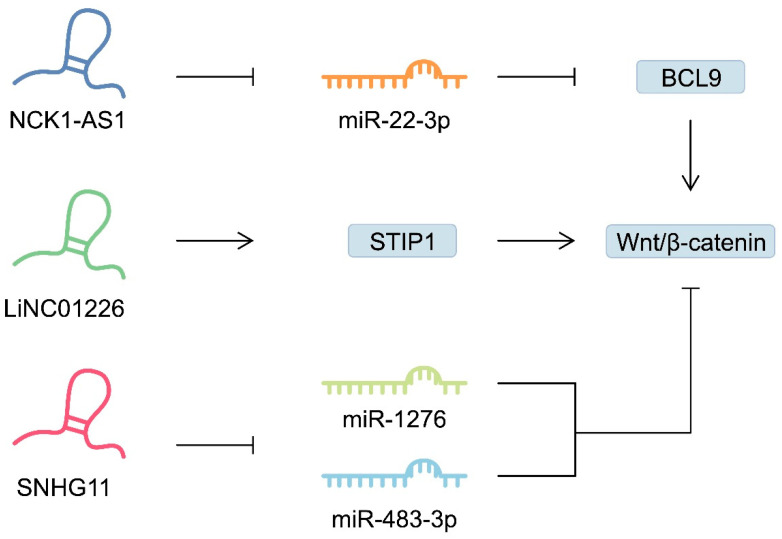
The involvement of selected long non-coding RNAs (lncRNAs) in regulating the activity of the Wnt/β-catenin signalling pathway.

Furthermore, lncRNAs regulate the Wnt/β-catenin pathway, enhancing cell proliferation, migration and invasion [173]. Liu et al. [174] investigated the function of long non-coding RNA colon cancer-associated transcript 5 (CCAT5) in promoting GC progression. They showed that CCAT5 may be a key promoter of GC growth and metastasis and a potential independent prognostic factor for GC. Mechanistically, lncRNA CCAT5 binds to the C-terminal domain of STAT3, blocking the Src homology 2 domain-containing protein tyrosine phosphatase 1 (SHP-1). Through this mechanism, it prevents the dephosphorylation of STAT3 at position Y705, leading to the activation of STAT3 and its translocation to the nucleus, thereby accelerating the progression of tumourigenesis. Guan et al. [175] confirmed enhanced activation of Wnt/β-catenin signalling in GC through the NCK1-AS1/miR-22-3p/BCL9 axis. The researchers showed that increased BCL9 levels in GC cells increased the stability of β-catenin and the activation of the signalling pathway. In addition, NCK1-AS1 activated the pathway through the miR-22-3p/BCL9 axis, leading to increased BCL9 expression. As a result of increased BCL9 expression, the corresponding target genes of the Wnt/β-catenin pathway are activated, which promotes oncogenic processes such as proliferation, invasion and metaplasia of GC. Another potential oncogene in GC is LINC01226, as it promotes GC progression by enhancing stress-induced phosphoprotein 1 (STIP1) translocation from the cytoplasm to the nucleus and the stabilisation of β-catenin protein [176]. Furthermore, LINC00323 may be a potential biomarker and prognostic factor indicative of OS and PFS [177]. Other lncRNAs dysregulated in GC that interact with the Wnt/β-catenin cascade include SNHG11 [178], SNHG22 [179], LINC01503 [180], LINC01314 [181], LINC01225 [182], LINC01606 [183], LINC00665 [184], and testis-specific transcript Y-linked 15 (TTTY15) [185], amongst oteros. These molecules also show significant correlations with clinical parameters. For example, low expression of SNHG22 was associated with a higher 5-year survival rate in patients with GC [179]. Furthermore, elevated expression of LINC01225 correlated positively with lymph node metastasis, TNM stage and invasion depth [182]. Figure 4 schematically demonstrates the involvement of selected lncRNAs in GC by regulating the Wnt/β-catenin pathway.

### 3.3. Circular RNAs—Involvement in the Pathogenesis of Gastric Cancer and Their Role as Biomarkers

Numerous studies have shown that circRNAs are expressed not only in healthy eukaryotic cells but also in tumour tissue. Similar to miRNAs and lncRNA, analyses of GC tissues have shown that circRNAs can be up- or downregulated. GC is a neoplasm that does not have any specific biomarker that could help detect the disease quickly and at an early stage. CEA and CA19-9, used commonly for gastrointestinal malignancies, have limited utility in diagnosing GC due to their low specificity and sensitivity [186,187]. Therefore, endoscopy, usually followed by tissue biopsy, is a procedure of choice. Histopathological examination is crucial to confirm or exclude the GC diagnosis [188]. Moreover, the biopsied tissue can be evaluated for tumour biomarkers. A perfect diagnostic biomarker is characterised by its organ specificity.

hsa_circ_0001821 is expressed in different cancer tissues, including breast cancer, lung cancer, colorectal cancer and GC. Its expression is increased in colorectal tissues and decreased in tissues and blood from patients with GC compared with healthy controls. Kong et al. [189] showed that hsa_circ_0001821 expression correlated negatively with GC tumour depth and lymph node metastasis. There was no correlation for other tumours, which supports the idea of hsa_circ_000182 organ specificity. Moreover, the ROC curve analysis showed that plasma hsa_circ_000182 was a more sensitive GC marker (AUC = 0.872, 95% CI 0.767–0.977) than CEA (AUC = 0.839, 95% CI 0.740–0.937), CA125 (AUC = 0.742, 95% CI 0.613–0.871) and CA19-9 (AUC = 0.771, 95% CI 0.649–0.893). Combining hsa_circ_000182 with those markers resulted in even greater sensitivity and specificity in detecting GC (AUC = 0.933). Considering these results, hsa_circ_000182 could be useful as a sensitive and specific biomarker of GC.

The expression of hsa_circ_0000745 differs in patients with GC and healthy controls. Its levels are much lower in gastric tissue and plasma samples from patients with GC. Tissue expression correlated negatively with tumour differentiation. However, no such association was observed in terms of TNM stage, tumour size, lymph node metastasis and CEA levels. hsa_circ_0000745 plasma levels correlated positively with the TNM stage [190]. Furthermore, hsa_circ_0000745 showed a higher sensitivity for GC screening than CEA (85.5% and 30%, respectively, with an AUC of 0.683 and 0.734, respectively) [190,191]. Combining plasma hsa_circ_0000745 and CEA levels increased the diagnostic value (AUC = 0.775). hsa_circ_001888 was downregulated in GC tissues and plasma samples from patients with GC and correlated negatively with histological grading [192].

hsa_circ_0000467 was significantly overexpressed in GC tissues, cell lines and plasma compared with the control group [193]. Its expression correlated positively with the TNM stage. Low hsa_circ_0000467 expression resulted in a better prognosis. The authors also compared hsa_circ_0000467 to other tumour markers, namely CEA and CA72-4. hsa_circ_0000467 had higher diagnostic value than CEA and CA72-4 (AUC = 0.790, 0.560 and 0.670, respectively).

The expression of circRNAs can differ between GC tissues and bodily fluids. Li et al. [194] reported downregulated CDR1as expression in GC tissues and its association with tumour size and neural invasion. However, it was upregulated in plasma, which in turn correlated with lymph nodes metastasis.

We may predict the patient’s clinical state by measuring levels of circRNAs because they are usually correlated with clinicopathological factors. Low expression of hsa_circ_0006633 correlated negatively with distal metastasis [195]. Similarly, low expression of hsa_circ_0001017 and hsa_circ_0061276 significantly decreased OS [196]. hsa_circ_002059, hsa_circ_0014717, hsa_circ_0000190, hsa_circ_0003159 and hsa_circ_0000181 have also been correlated with distant metastasis [197,198,199,200,201].

The most relevant benefit of a diagnostic biomarker is its possible ability to detect the disease at an early stage. Early gastric cancer (EGC) is defined as carcinoma limited to the stomach mucosa and/or submucosa regardless of the lymph node status [202]. Currently, endoscopy is the standard tool to detect GC. However, this method is limited as the EGC lesion is usually macroscopically undetectable [203]. Moreover, the quality of the examination is highly dependent on the experience, skills and awareness of the endoscopist. Finding a simple and non-invasive screening method could be beneficial for diagnosing GC at early stages. Lu et al. [204] investigated whether EGC could be detected using peripheral blood markers. A ribosomal protein, RPL6, was overexpressed in GC. By contrast, RPL6 was downregulated in EGC. The authors found that hsa_circ_0006848, a circRNA present in the plasma, is related to the miR-329-5p/RPL6 axis, which is known to be connected to GC. The hsa_circ_0006848 plasma levels were lower in patients with the EGC compared with the healthy controls and patients with advanced gastric cancer (AGC). Moreover, the hsa_circ_0006848 plasma levels correlated negatively with the tumour size and histological grade. The standard serum tumour markers, such as CEA and CA19-9, are not exclusive for GC. Thus they present low sensitivity and specificity when diagnosing GC, especially EGC [205]. The CEA and CA19-9 plasma levels in patients with EGC were normal in that study group, whereas the hsa_circ_0006848 levels were significantly lower; thus, hsa_circ_0006848 might have good diagnostic value (AUC = 0.733) [204]. Furthermore, Shao et al. [206] reported downregulated hsa_circ_0001895 expression in GC and precancerous samples. The expression level correlated negatively with histological grade. The sensitivity and specificity of hsa_circ_0001895 were 67.8% and 85.7%, respectively, and the AUC was 0.792, indicating that this circRNA is a potential marker of EGC. Another useful asset of a biomarker is its ability to detect the recurrence of a disease. Fang et al. [207] discovered that patients with a renewal of GC presented increased expression of hsa_circ_0058246. Thus, it may serve as a good diagnostic marker of recurrence.

GC biomarkers can be isolated not only from plasma samples but also from other bodily fluids, such as gastric juice. Gastric juice analysis is a more invasive procedure than blood sampling as it requires inserting a nasogastric tube to aspirate the fluid. However, this method may be useful in diagnosing *H. pylori* infection or lesions of the gastric mucosa that are not macroscopically detectable [208]. Ummarino et al. [209] evaluated whether gastric juice analysis could minimise the number of performed biopsies or identify patients in whom biopsies should be performed. They reported that histological lesions are related to the pH of the gastric juice: the number of lesions increases proportionally with the pH. Therefore, gastric juice analysis is a sensitive indicator of gastric health. Performing biopsies (two in the antrum and two in the fundus) in all patients with hypochlorhydria resulted in a much better rate of detecting gastric mucosal lesions. Because pH measurement is a simple test that helps to indicate patients with probable preneoplastic conditions or GC, additional detection of circRNAs may enhance its benefits. For example, the hsa_circ_000780 levels in gastric juice and its expression in gastric tissue were measured in samples from patients with EGC, AGC, chronic nonatrophic gastritis and chronic atrophic gastritis as the control group [210]. hsa_circ_000780 was significantly downregulated in the GC group, similar to the gastric juice samples. However, there were no significant differences in the hsa_circ_000780 levels between the EGC and AGC groups. The low expression also correlated positively with tumour size, T stage, venous invasion and CEA and CA19-9 levels. These findings suggest that identifying hsa_circ_000780 during gastric juice analysis could be useful in screening for GC, including EGC. Another circRNA that can be found in gastric juice is hsa_circ_0014717 [198].

Various studies have demonstrated the vital role of circRNAs in the pathogenesis of GC. In addition, these molecules could be used as novel prognostic biomarkers to identify patients who are more likely to have a recurrence or a poor prognosis. circRNAs interact with multiple members of the signalling pathways that ultimately promote cell proliferation and inhibit apoptosis, which drive tumourigenesis. Studies have determined that circRNAs affect the PI3K/Akt, Wnt/β-catenin, MAPK, VEGF and TGF-β signalling pathways. Pan et al. [211] demonstrated that ciRS-7 attenuated the inhibitory effects of miR-7 on the PTEN/PI3K/AKT pathway. miR-7 overexpression reduced PI3K and AKT phosphorylation, changes that were reversed by ciRS-7, suggesting its oncogenic potential. Similarly, circAKT3 increased the expression of PIK3R1, which plays a role in the process of cisplatin resistance in GC. Mechanistically, circRNAs could induce this effect by sponging miR-198 [212]. By contrast, circNRIP1 suppressed the PI3K/Akt signalling by sponging miR-149-5p [213].

Regarding the Wnt/β-catenin cascade, Zang et al. [214] revealed that circEIF4G3 suppressed GC growth by decreasing β-catenin protein expression, which consequently reduced the expression of c-Myc and cyclin D1. This circRNA acted through the miR-4449/SIK1 axis, which ultimately disturbed the interaction between β-catenin and TBL1/TBLR1 complex (its coactivators). circ-HN1 also modulates the Wnt/β-catenin pathway. A luciferase reporter verified the interaction between circ-HN1, miR-485-5p and glycogen synthase kinase-3 (GSK3A). circ-HN1 acted as a sponge for miR-485-5p, leading to an increased expression of GSK3A, a member of the Wnt/β-catenin signalling pathway, and tumour formation [215]. circPDIA4 interacted with ERK1/2, enhancing its phosphorylation. Mechanistically, it interfered with the association between DUSP6, a negative regulator of ERK. Overexpressed CircPDIA4 increased ERK1/2 phosphorylation in GC cells, thus leading to hyperactivation of the MAPK signalling cascade [216]. Lu et al. [217] demonstrated that circ-RanGAP1 acted as a sponge of miR-877-3p, a molecule that targets and inhibits VEGFA. Consequently, circ-RanGAP1 stimulated the expression of VEGFA. Its overexpression augmented the invasion and migration of GC cells, findings that suggest circ-RanGAP1 enhances GC progression. Moreover, circWNK1 inhibited TGFβ signalling by acting as a ceRNA for SMAD7. It sequestered miR-21-3p, which reversed its inhibitory effect on SMAD7. Because SMAD7 inhibits the TGFβ pathway, it also inhibits GC progression [218]. He et al. [219] reported an interaction between circ_0006282 and miR-155. Knockdown of this circRNA resulted in the upregulation of miR-155 and subsequent inhibition of FBXO22 expression, which negatively affected the progression of GC.

Importantly, circRNAs could also serve as prognostic biomarkers. High circPDIA4 levels have been linked to poor survival outcomes in patients with GC. In mouse xenograft models, higher circPDIA4 expression was associated with metastasis. Both PFS and OS were negatively affected by higher circPDIA4 levels [216].

According to Zang et al. [214], circEIF4G3 suppresses GC growth. Downregulation of this molecule was associated with worse clinical outcomes, such as survival and TNM stage. Additionally, reduced expression of circEIF4G3 was connected to the presence of malignant blood vessels.

circWNK1 is a promising prognostic biomarker for GC. Its expression was considerably downregulated in cancer tissues, which was associated with more advanced tumour stages and worse histological grades in patients with GC. Furthermore, patients with downregulated circWNK1 had a substantially worse 3-year survival rate [218].

Higher expression of circ-RanGAP1 has been linked with more aggressive features, such as larger tumour size, advanced TNM stage and lymph node metastasis. Multivariate Cox regression revealed that upregulated circ-RanGAP1 was an autonomous prognostic factor for poor survival [217]. Similarly, upregulated circNRIP1 was associated with worse clinical parameters in the GC population [213].

Other circRNAs suggested to act as promising prognostic biomarkers include circFAT1(e2) [220], circPVT1 [221], ciRS-7 [211], hsa_circ_0001368 [222], circLPAR1 [223] and hsa_circ_0015286 [224], amongst others. circRNAs can also serve as markers of immune modulation, suggesting different responses to immunotherapy. For example, knockdown of circDLG1 enhanced the CD8+ T cell population but suppressed myeloid-derived suppressor cells (MDSCs). Mechanistically, circDLG1 acts as a sponge that binds miR-141-3p, which stimulates the expression of CXCL12. Therefore, it might promote resistance to anti-programmed cell death protein 1 (PD-1) therapy [225]. Table 4 summarises the impact of circRNAs on clinical parameters in GC.

## 4. Conclusions and Future Perspectives

Numerous studies have implicated ncRNAs—miRNAs, lncRNAs and circRNAs—in the pathogenesis of GC. Importantly, dysregulated expression of these molecules could be used clinically as diagnostic and/or prognostic biomarkers. There have been conflicting results amongst studies that have evaluated miRNAs as biomarkers. These differences could result from different characteristics of the study groups, data-processing methods or statistical methods [226]. To increase the quality of studies investigating potential diagnostic biomarkers, various material sources could be used. For example, the data could be obtained from one of the large, publicly available databases, such as TCGA, and/or from cell lines and clinical samples. Furthermore, validation cohorts may strengthen the obtained results. Importantly, some differences could result from different reference genes used in the analyses to analyse the reliability of quantitative reverse transcription–polymerase chain reaction results. Importantly, the use of different reference genes may significantly change the results [22]. ncRNAs are exciting and promising biomarkers. Nevertheless, future clinical trials should precisely evaluate the true diagnostic potential of particular molecules, as they can be dysregulated not only in malignancies but also in other benign diseases. Furthermore, a combination of ncRNAs with classical clinical parameters and radiological methods or the formation of ncRNA panels composed of several molecules could significantly enhance their diagnostic potential. Knowledge about the involvement of ncRNAs in signalling pathways that contribute to tumourigenesis is crucial to understand the regulatory processes associated with cellular proliferation, apoptosis, metastasis and treatment resistance. In this paper, we focused on the PI3K/AKT, MAPK and Wnt/β-catenin pathways. However, ncRNAs regulate GC tumourigenesis by mediating the activity of other molecules and pathways as well. For example, MACC1-AS1 is a lncRNA transcribed from an intron of the *MACC1* gene, which mediates the expression of *MET*. MACC1-AS1 was found to regulate the progression of various malignancies, including GC. Studies demonstrated that this molecule regulates metabolic pathways, which could be associated with GC progression and treatment resistance [227,228]. Therefore, ncRNA mediates multiple processes that can promote tumour formation or treatment resistance. Future studies should also investigate the influence of commonly used drugs on ncRNA expression in GC. For instance, esomeprazole was found to affect the ncRNA interaction network and impact GC progression [229]. Understanding these mechanisms may be involved with novel management strategies that could translate into improved treatment outcomes in the future.

## Figures and Tables

**Table 2:** Summary of selected micro RNAs (miRNAs) associated with clinical parameters in gastric cancer.

MicroRNA	Clinical Parameter	References
miR-23a-3p, miR-495, miR-181d, miR-30e-3p, miR-489, miR-21, miR-718, miR-575, miR-616-3p, miR-589, miR-95-3p, miR-140-5p, miR-188-5p, miR-520f-3p, miR-501-5p	Overall Survival	[70,71,72,73,74,86,87,95,96,100,105,106,132,133,137,139]
miR-495, miR-21, miR-140-5p, miR-520f-3p	Disease-Free Survival	[71,86,132,133]
miR-489	Progression-Free Survival	[74]
miR-21, miR-582, miR-592, miR-141, miR-633, miR-140-5p, miR-188-5p	Lymph Node Metastasis	[87,107,117,119,121,132,139]

**Table 3:** A summary of selected studies that have investigated the role of long non-coding RNAs (lncRNAs) as diagnostic biomarkers for gastric cancer.

lncRNA	Source	Expression in Gastric Cancer	Diagnostic Potential (AUC)	Reference
PCGEM	Plasma	Upregulated	0.750	[146]
H19	Plasma	Upregulated	0.838	[147]
HULC	Serum	Upregulated	0.888	[148]
HOTAIR	Plasma	Upregulated	0.944	[153]
GNAQ-6:1	Serum	Downregulated	0.736	[154]
PCSK2-2:1	Serum	Downregulated	0.896	[155]
HULC	Plasma	Upregulated	0.650	[156]
ZNFX-AS1	Plasma	Upregulated	0.850	[156]
CUDR LSINCT-5 PTENP1	Serum	Upregulated	0.829	[157]
AFAP1-AS1	Tissue	Upregulated	0.893	[158]
FBXO18-AS	Tissue	Upregulated	0.822	[159]
BOK-AS1	Tissue	Upregulated	0.737	[160]
FAM215A	Tissue	Upregulated	0.716	[160]
FEZF1-AS1	Tissue	Upregulated	0.712	[160]
SNHG15	Tissue	Upregulated	0.744	[161]
FOXD2-AS1	Tissue	Upregulated	0.965	[163]
LINC01235	Tissue	Upregulated	0.916	[163]
RP11-598F7.5	Tissue	Upregulated	0.963	[163]

**Table 4:** A summary of selected circular RNAs (circRNAs) associated with clinical parameters in gastric cancer.

CircRNA	Clinical Parameter	References
CircPDIA4, circWNK1, circNRIP1, circFAT1(e2), circPVT1, circRNA ciRS-7, Hsa_circ_0015286, circLPAR1, circEIF4G3, Hsa_circ_0015286	Overall Survival	[211,212,213,215,216,220,221,223,224]
circNRIP1, circPVT1	Disease-Free Survival	[213,221]
Circ-RanGAP1, circNRIP1, circLPAR1, Hsa_circ_0015286	Lymph Node Metastasis	[213,214,223,224]
circWNK1, Circ-RanGAP1, circEIF4G3, circLPAR1, Hsa_circ_0015286	TNM Stage	[214,216,223,224]
CircEIF4G3, circLPAR1	Venous invasion	[212,223]
CircPDIA4,	Progression-Free Survival	[215]
Circ-RanGAP1, CircEIF4G3, circNRIP1, circLPAR1, Hsa_circ_0015286	Tumor Size	[212,213,214,223,224]
CircAKT3	Cisplatin Resistance	[218]
circDLG1	Resistance to anti-PD-1therapy	[225]

PD-1—programmed cell death protein 1.
